# Experimental Characterization and Numerical Assessment of Cu-Al-Be Shape Memory Alloys for U-Shaped Flexural Plates

**DOI:** 10.3390/ma19122617

**Published:** 2026-06-17

**Authors:** Catalina Santibañez, Ramiro Bazáez, Luis Pérez, Yessica L. Avila-Avila, Gabriel Lara-Rodríguez

**Affiliations:** 1 Departamento de Ingeniería Mecánica, Universidad Técnica Federico Santa María, Av. España 1680, Valparaíso 2390123, Chileluis.perez@usm.cl (L.P.); 2 Departamento de Obras Civiles, Universidad Técnica Federico Santa María, Av. España 1680, Valparaíso 2390123, Chile; 3 Instituto de Investigación en Materiales, Universidad Nacional Autónoma de México (IMM-UNAM), Circuito Exterior S/N, Ciudad Universitaria, Ciudad de Mexico 04510, Mexico; yavila@pceim.unam.mx (Y.L.A.-A.); laragab@unam.mx (G.L.-R.)

**Keywords:** shape memory alloys (SMA), Cu-Al-Be (CAB), U-shaped device, superelasticity, seismic energy dissipation

## Abstract

This study presents an experimental characterization and numerical assessment of Cu–Al–Be (CAB) shape memory alloys (SMAs) for potential applications in U-shaped flexural plate (UFP) seismic dampers. Six alloy compositions were evaluated through monotonic tensile tests, ASTM F2516 superelastic protocols, and increasing-amplitude cyclic loading to identify the material exhibiting stable superelastic behavior at room temperature. Among the tested materials, alloy CAB4.76-A showed the most favorable response, with high transformation stress, stable pseudoelastic behavior, and strain recovery exceeding 95% for strains up to 2.5%. A phenomenological finite element model based on the Auricchio constitutive formulation was calibrated using experimental data within the validated strain range (ε ≤ 0.025), showing good agreement in stiffness and stress prediction. The calibrated model was subsequently applied to simulate the response of a UFP device under orthogonal cyclic loading. The results indicate a strong dependence on loading orientation due to coupled bending–torsion effects, with the 90° direction exhibiting significantly higher strength and energy dissipation capacity. Comparison with analytical formulations originally developed for steel UFPs showed that these expressions provide approximate estimates when applied to SMA-based devices. The results suggest that Cu–Al–Be alloys are a promising alternative for UFP applications, while highlighting the importance of loading orientation and the need for future experimental validation at a device scale.

## 1. Introduction

Structural damage caused by recent earthquakes has emphasized the need for resilient infrastructure capable of maintaining functionality after extreme events. In this context, seismic protection devices that combine energy dissipation with self-centering capability have emerged as effective solutions for reducing residual damage and enhancing post-earthquake resilience [1,2,3,4,5].

**Figure 1:**
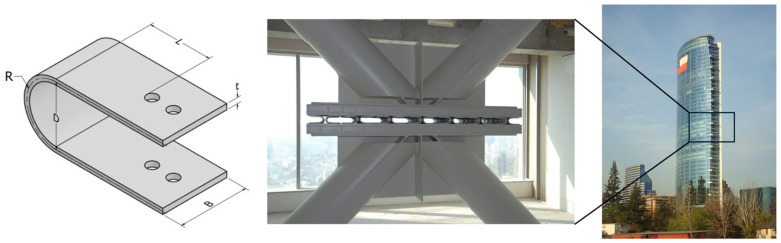
U-Shaped Flexural Plates implemented on the Titanium Building in Chile.

Among metallic seismic dampers, U-Shaped flexural plates (UFPs) are particularly attractive due to their geometric simplicity, given by their width: B, effective length: L, diameter: D, and thickness: t, low fabrication cost and stable hysteretic response under severe cyclic loading [6]. These devices were first proposed by Kelly et al. [7] and have traditionally been manufactured from steel. UFPs dissipate energy through controlled flexural deformation and have been successfully implemented in both new and retrofitted structures [8,9,10,11], as shown in Figure 1. Subsequent research explored more complex geometric configurations [12,13] and developed analytical models to estimate stiffness and strength [6,14,15,16]. However, a key limitation of steel-based UFPs is the accumulation of plastic deformation, which leads to permanent residual displacements after strong seismic events.

In recent years, increasing attention has been directed toward smart materials capable of providing structural recentering. Shape memory alloys (SMAs) have emerged as promising candidates due to their ability to recover large deformations through reversible phase transformations between martensite and austenite. This transformation enables energy dissipation while minimizing permanent residual strain [3,4,17,18,19,20,21,22,23,24]. Within the family of SMAs, three main groups stand out: NiTi-based alloys (Nitinol), ferrous alloys (Fe-SMA) and copper-based alloys (Cu-SMA). NiTi alloys are the most studied and widely used due to their excellent superelasticity, thermal stability and fatigue resistance [5,19]. However, their high cost and processing complexity may limit their large-scale application in civil structures. Qian et al. [25] noted that Cu-based SMA production costs can be reduced to approximately 10% of NiTi, making them economically attractive for large-scale structural applications. Ferrous alloys (such as Fe–Mn–Si) offer low cost and good weldability, but their recovery capability is limited and strongly dependent on specific heat treatments [26,27]. Experimental tensile and cyclic tests comparing NiTi, Cu-Al-Ni and Fe-Mn-Si bars confirm that NiTi and Fe-based alloys show higher transformation stresses and energy dissipation, while NiTi stands out for recentering with virtually no residual strain [28]. In contrast, copper-based alloys such as Cu–Al–Be, Cu–Zn–Al and Cu–Al–Mn offer better machinability and lower cost, with adjustable transformation temperatures [29,30].

Recent studies have demonstrated that Cu–Al–Be alloys exhibit promising superelasticity, thermal stability, and cyclic resistance [29,31]. Medina et al. [32] identified heat treatments that optimize the superelastic response of Cu-Al-Be wires, and their application in self-centering systems has been validated numerically [33]. Saeedi et al. [34] demonstrated that Cu–Al–Be SMA bars combined with high-damping rubber bearings reduce maximum displacements by up to 79% under near-fault ground motions, validating their self-centering potential in seismic isolation [35]. However, their real structural implementation in energy dissipation devices remains limited.

Despite extensive research on U-shaped flexural plates (UFP) and the increasing interest in shape memory alloys for seismic applications, most existing studies have focused on NiTi or Fe-based alloys and primarily on unidirectional loading conditions [19,21,26,35]. In contrast, the use of Cu–Al–Be alloys in UFP devices remains largely unexplored, particularly considering their lower cost and promising superelastic properties. Furthermore, limited studies combine experimental material characterization with device-level numerical assessment. Therefore, the objective of this study is to experimentally characterize different Cu–Al–Be alloy compositions and to numerically assess their potential application in UFP devices. The study includes (i) material-level experimental testing to identify a suitable composition for superelastic alloys, (ii) calibration of a finite element model based on experimental data, and (iii) numerical evaluation of a UFP device under orthogonal cyclic loading. The numerical results are further compared with existing formulations for estimating UFP stiffness and yield force.

## 2. Experimental Program

This study presents the experimental evaluation of six variants of Cu–Al–Be (CAB) shape memory alloys (SMAs). The selected alloy compositions were designed to cover a range of transformation temperatures, aiming to identify the composition that exhibits stable behavior, the highest mechanical strength and the lowest residual strain for implementation in a UFP device.

The alloys are identified using a two-term nomenclature. The first term denotes the beryllium (Be) content per mille (wt‰), and the second indicates the expected phase during testing (A: austenite, M: martensite, AM: mixed phase). For example, in CAB4.40-AM, “4.40” represents the Be content, while “AM” indicates the coexistence of austenite and martensite in the microstructure.

### 2.1. Casting and Processing of Cu–Al–Be Alloys

The shape memory alloys were prepared using electrolytic copper (99.99%), high-purity aluminum (99.99%) and a Cu–4%Be master alloy. Melting was carried out in a radio-frequency induction furnace using an isostatic graphite crucible under a high-purity argon atmosphere (99.99%). Through an in-situ casting process into solid steel molds, ingots with approximate dimensions of 65 mm in width, 130 mm in length and 15 mm in thickness were produced.

Subsequently, the ingots were hot-rolled at 800 °C using a 5-inch two-high rolling mill, reducing their thickness from 15 mm to 5 mm (33% reduction). The resulting slabs (65 mm × 30 mm × 5 mm) were subjected to a homogenization heat treatment at 850 °C for 20 min and quenched in boiling water at ≈90 °C for 20 min to stabilize the microstructure and homogenize the phase distribution.

### 2.2. Alloy Characterization and Classification

**Figure 2:**
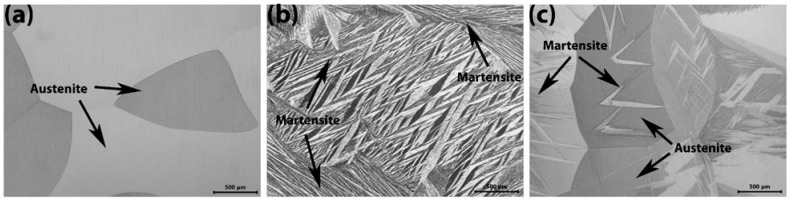
Optical micrographs (5X) of Cu–Al–Be alloys: (**a**) CAB4.76-A (austenite); (**b**) CAB4.10-M (martensite); (**c**) CAB4.40-AM (mixed phase).

For microstructural characterization of the alloys, samples measuring 10 mm × 10 mm × 5 mm were prepared. The surfaces were polished with 600, 1000 and 2000 grit SiC papers, followed by alumina (Al_2_O_3_) polishing to a mirror finish. The microstructure was revealed by chemical etching via immersion for 5-s in a reactive solution composed of FeCl_3_ (5 g, 8.51%), 37.5% HCl (16 mL, 32.51%) and ethanol (44 mL, 59.08%). The micrographic observations, Figure 2a, show austenitic phase grain boundaries, nucleating preferentially at a triple point boundary and growing gradually into the grains. It can also be observed that the grain size is large, measuring approximately 1.5 to 2 mm, as determined by the ASTM E112 standard [36]. Figure 2b shows the typical microstructure of the martensitic phase, formed by the nucleation of martensite variants within the grains of the austenitic phase. Finally, Figure 2c illustrates the mixed-phase condition, highlighting the coexistence and interaction between the austenitic and martensitic phases.

**Figure 3:**
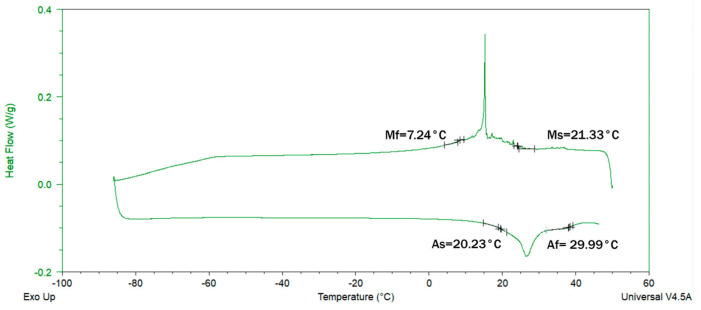
DSC thermogram of sample CAB4.65-AM.

**Figure 4:**
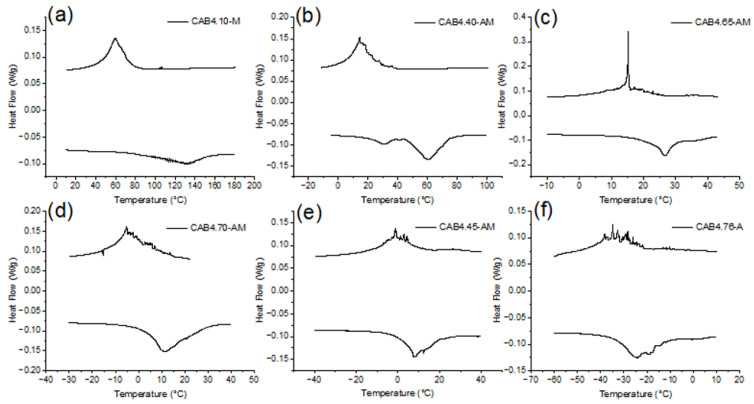
DSC comparative thermograms of the Cu–Al–Be SMA. (**a**) CAB4.10-M. (**b**) CAB4.40-AM. (**c**) CAB4.65-AM. (**d**) CAB4.70-AM. (**e**) CAB4.45-AM. (**f**) CAB4.76-A.

Transformation temperatures were determined by differential scanning calorimetry (DSC) using a Q2000 apparatus (TA Instruments, New Castle, Delaware, USA), in accordance with the ASTM F2004-24 standard [37]. Samples weighing between 100 and 150 mg were subjected to heating and cooling cycles from −80 °C to 250 °C at a controlled rate of 10 °C/min under a nitrogen atmosphere. The transformation temperatures, namely martensite start (*M_s_*), martensite finish (*M_f_*), austenite start (*A_s_*), and austenite finish (*A_f_*), were identified from the resulting thermograms using the tangent-line intersection method as prescribed by ASTM F2004-24. This method defines each transformation temperature as the intersection point between the linearly extrapolated baseline and the tangent drawn at the steepest slope of the corresponding exothermic (cooling) or endothermic (heating) peak. The analysis was performed with TA Universal Analysis software, version 4.5A (TA Instruments), which enables the graphical application of this method directly on the recorded thermograms. A representative DSC thermogram, depicting both the heating and cooling cycles along with the graphical identification of the four transformation temperatures, is shown in Figure 3, while the DSC for all the specimens is shown in Figure 4.

The chemical composition of Cu and Al was determined by energy-dispersive X-ray spectroscopy (EDS) using a scanning electron microscope (SEM), with a minimum of five measurement points per sample; reported values represent averages with an estimated uncertainty of ±0.1–0.2 wt.%. Be could not be quantified by EDS due to its low atomic number, nor reliably by ICP analysis due to matrix shielding effects associated with its light atomic mass. Thus, the Be contents reported correspond to nominal design values. The chemical composition and transformation temperatures obtained from DSC analysis are presented in Table 1.

The thermal characterization shows a strong sensitivity of transformation temperatures to compositional variations in the CAB alloys. Beryllium shows a pronounced effect, as expected for Cu–Al–Be systems. At nearly constant Al content (~11.70 wt.%), a small increase of 0.03 wt.% Be (from CAB4.10-M to CAB4.40-AM) leads to a significant reduction in Ms of 44.5 °C. A similar trend is observed at higher Al levels (~11.99 wt.%), where an increment of only 0.011 wt.% Be decreases Ms by 44.8 °C, shifting the transformation range from near-ambient (21.3 °C) to sub-zero temperatures (−23.5 °C). These results confirm the high sensitivity of transformation temperatures to minor Be variations. The pronounced effect of Be on the transformation temperatures of CAB alloys is associated with its influence on the stability of the parent β-austenite phase. Small additions of Be modify the thermodynamic balance between austenite and martensite, increasing the stability of the β phase. As a result, a larger undercooling is required to initiate the martensitic transformation, leading to a significant reduction in *M_s_*. Furthermore, Be promotes atomic ordering and affects the lattice parameters of the parent phase, which alters the driving force for transformation. Consequently, even minor variations in Be content can produce substantial shifts in transformation temperatures, as observed in the present experimental results. In contrast, the influence of Al, although relevant, requires larger compositional changes to produce comparable shifts. At approximately constant Be (~0.47 wt.%), an increase of 1.40 wt.% Al reduces *M_s_* by about 23 °C. This indicates that, within the studied compositional range, Be is more effective in tuning transformation temperatures. The experimental results for *M_s_* were compared with the empirical Equation (1) proposed by [38].

(1) $$M_{s}\left({}^{\circ}C\right)=1245-71\left(Al\;wt.\%\right)-893(Be\;wt.\%)$$

**Figure 5:**
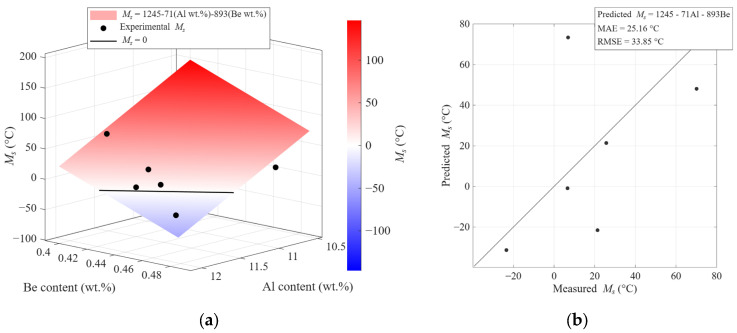
Effect of Al and Be content in *M_s_*. (**a**) 3D representation, (**b**) predicted vs measured *M_s_*.

As shown in Figure 5a, the experimental data and the model reproduce the overall shift in *M_s_* due to CAB composition. However, a quantitative assessment based on mean absolute error (MAE) and root mean square error (RMSE), presented in Figure 5b, reveals deviations between predicted and experimental values. These differences indicate that, although composition is the primary design parameter, transformation temperatures are also affected by processing-related factors such as heat treatment, quenching conditions, and grain size [39]. From an engineering perspective, the control of transformation temperatures is critical for performance. Alloys with *A_f_* below service temperature are expected to exhibit superelastic behavior, enabling reversible strain and energy dissipation under mechanical loading. In contrast, alloys with higher transformation temperatures remain partially or fully martensitic during operation, limiting their recoverable deformation and shifting their response toward shape memory behavior or phase coexistence. Accordingly, the alloys were classified based on their phase state under testing conditions (T ≈ 10 °C), providing a practical framework for selecting compositions suitable for structural applications requiring superelastic performance.

### 2.3. Specimen Geometry and Experimental Configuration

**Figure 6:**
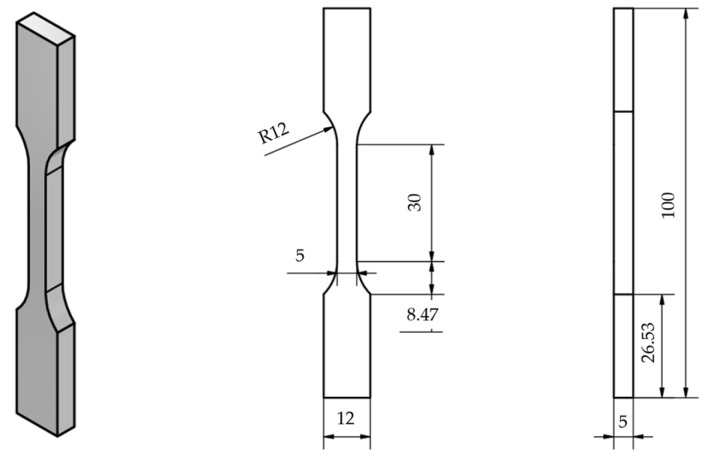
Dimensions of the specimen used in the tensile tests.

The tensile specimens were designed with a uniform geometry to facilitate fabrication and ensure strain localization within the gauge section. The specimens were machined directly from the hot-rolled slabs, maintaining a constant thickness of 5 mm and a total length of 100 mm. The gripping sections measure 26.53 mm, while the central gauge region has a length of 30 mm. The complete geometry is illustrated in Figure 6.

Experimental tests were conducted using an MTS 810 universal testing machine equipped with hydraulic grips, a 100 kN load cell and a linear variable differential transformer (LVDT), allowing the application of axial loads and deformations with high precision. The system was operated using an MTS controller connected to its dedicated software, through which parameters such as loading rate and control mode (force or displacement) were configured, ensuring stable conditions throughout the entire test.

**Figure 7:**
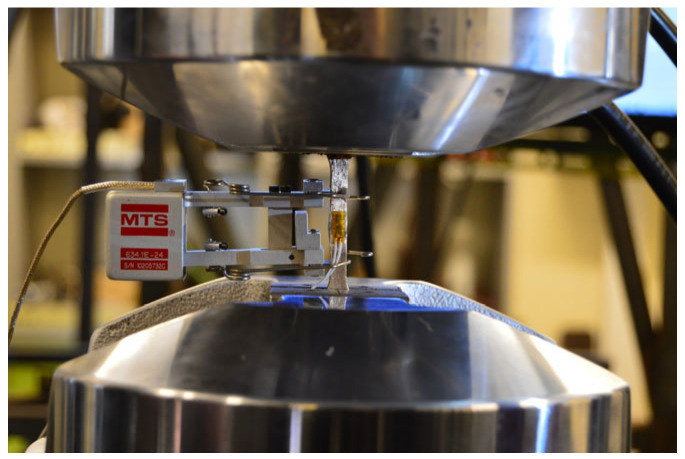
Experimental setup used for the tests in the MTS 810 universal testing machine, equipped with hydraulic grips and an axial extensometer.

An axial extensometer (MTS 634.11-54) with a 25 mm gauge length was attached directly to the gauge section to measure axial strain. In addition, strain gauges were bonded to the specimen surface to validate extensometer measurements and provide redundancy in strain acquisition. The complete experimental setup is shown in Figure 7.

The quasi-static tests were conducted at approximately 10 °C, consistent with the mean annual temperature in Chile (10 to 15 °C) and therefore representative of typical exterior service conditions for potential seismic devices in the region.

### 2.4. Testing Protocols

#### 2.4.1. Monotonic Test

A quasi-static uniaxial tensile test was performed under displacement control at a constant rate of 0.5 mm/min using the MTS testing machine. During the test, force and displacement data were continuously recorded, along with local strain measured by the axial extensometer. Each specimen was loaded until complete fracture. The objective of these tests was to determine ultimate stress and strain capacity without unloading the specimen.

#### 2.4.2. ASTM F2516 Test

The ASTM F2516 [40] standard protocol was applied to characterize the superelastic behavior of the alloys. The procedure consisted of loading the specimen to approximately 6% strain, as measured by the extensometer, unloading to zero force, and subsequently reloading the specimen to failure. All tests were performed under displacement control at a constant rate of 0.1 mm/min. This protocol complemented monotonic characterization by enabling the identification of characteristic phase-transformation stresses under controlled conditions.

Although ASTM F2516 was originally developed for superelastic NiTi alloys, its loading-unloading to failure sequence provides a standardized and reproducible framework for identifying characteristic transformation stresses in any superelastic SMA. In the absence of a dedicated standard for Cu-based SMAs, this protocol has been adopted in prior studies on Cu-based alloys as a reference characterization methodology. No modifications to the loading sequence were introduced; however, the target strain of approximately 6% was selected to encompass the expected superelastic transformation plateau of the Cu–Al–Be alloys tested, rather than being prescribed by the standard itself.

#### 2.4.3. Increasing-Amplitude Half-Cycle Test

Based on the results of the monotonic and ASTM F2516 tests, one alloy was selected for cyclic characterization. The test was performed under displacement control at a constant rate of 0.1 mm/min. The amplitudes were defined based on the characteristic phase-transformation strain of the material, estimated from the prior monotonic tests.

For each amplitude level, two unidirectional half-cycles were applied. Specifically, the specimen was loaded in tension up to the prescribed displacement and then unloaded to zero force without applying compression. The initial maximum displacement was 1 mm, and the amplitude was increased in increments of 0.5 mm per level until specimen fracture occurred. This protocol allowed evaluation of superelastic stability, strain accumulation, and energy dissipation under progressively increasing deformation demands.

## 3. Experimental Results

### 3.1. Monotonic Test Results

To interpret the mechanical behavior of SMAs under monotonic loading, analytical models widely used in literature were considered. According to Wilde et al. [41], three main regions can be identified in the stress–strain curve: the elastic austenitic phase, the stress-induced martensitic transformation region and the stable martensitic phase.

**Figure 8:**
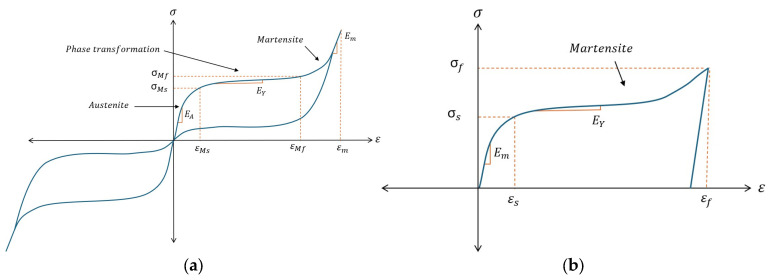
Conceptual Stress-Strain curves of SMA behavior: (**a**) superelasticity, (**b**) shape memory effect. Adapted from [18,27,41].

Complementarily, the schemes proposed by Qiang et al. [27], and Billah and Youssef [18] distinguish two characteristic functional mechanisms of SMAs: (i) the shape memory effect (SME), associated with thermally driven recovery of deformation upon exceeding the transformation temperatures, and (ii) superelasticity (SE), which occurs at temperatures above *A*_*f*_ and manifests as spontaneous strain recovery during mechanical unloading. Figure 8 presents both reference diagrams: (a) the stress–strain response exhibiting superelasticity and (b) the stress–temperature curve illustrating the shape memory effect.

**Figure 9:**
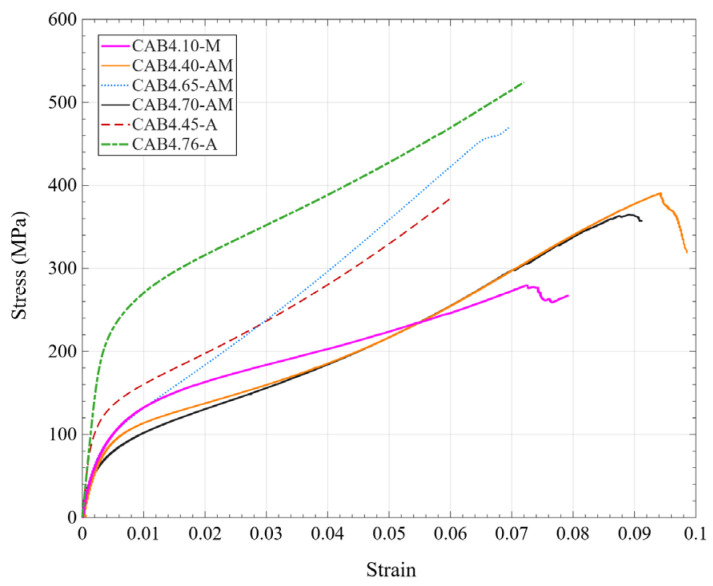
Stress–strain curves obtained from the monotonic tests.

The stress–strain curves obtained from the monotonic tests for all the specimens are shown in Figure 9. These curves allow a visual comparison of initial stiffness, characteristic transformation stresses and ultimate strain capacity among the different materials. The mechanical parameters extracted from these curves were based on the expected behavior (i.e., shape memory or superelasticity). For the initial martensitic or mixed phase alloys, primarily associated with the shape memory effect, the martensitic elastic modulus (*E*_*m*_), the modulus of linear pseudoelastic strain hardening at simple tension (*E*_*y*_), the characteristic start and finish transformation stresses (*σ*_*s*_ and *σ*_*f*_) and the corresponding strains ($\varepsilon_{s}$ and $\varepsilon_{f}$) were determined. In contrast, for the initial austenitic phase alloys, which exhibited superelastic behavior during the tests, the austenitic elastic modulus (*E*_*A*_), the slope during transformation (*E*_*y*_), the start and finish stresses of the superelastic transformation (*σ*_*Ms*_ and *σ*_*Mf*_), the ultimate stress (*σ*_m_) and the corresponding strains ($\varepsilon_{Ms}$, $\varepsilon_{Mf}$, and $\varepsilon_{m}$) were identified as summarized in Table 2.

By comparing the composition of the CAB alloys with the mechanical properties obtained from the monotonic tests (Table 2), it is observed that the mechanical response of the CAB SMAs is highly sensitive to slight variations in chemical composition. In general, increasing the Al content toward 12 wt.% and the Be content up to 0.476 wt.% led to a significant improvement in stiffness and strength. This trend is illustrated by the increase in elastic modulus from 16.9 GPa for CAB4.70-AM to 69.7 GPa for CAB4.76-A, accompanied by an increase in transformation stress from 71 MPa to 239 MPa. The higher Al concentration enhanced the stability of the ordered β phase, while Be addition contributed to microstructural refinement and strengthening, thereby increasing the stress required to induce martensitic transformation.

It should be noted that the *E_m_* values reported for martensitic and mixed-phase alloys (e.g., 16.9 GPa for CAB4.70-AM) do not represent true elastic moduli of the martensite phase. At T ≈ 10 °C, these compositions are near or within their transformation range; consequently, the initial slope of the stress–strain curve may incorporate contributions from variant reorientation, detwinning, or the onset of stress induced transformation. These values should therefore be interpreted as effective tangent stiffness values under the specific test conditions, and their temperature dependence should be considered when extrapolating to other service temperatures.

From a functional perspective, the results indicate that the combination of composition plays a critical role in tailoring the mechanical performance of CAB SMAs. Alloys with lower elastic modulus and lower transformation stresses may be more suitable for applications requiring easier martensitic transformation, whereas alloys such as CAB4.76-A, with superior mechanical strength and stiffness, are more attractive for structural applications where higher load-bearing capacity is required. However, these observations are based solely on the mechanical characterization performed at the testing temperature considered in this study (T ≈ 10 °C). Since the functional behavior of shape memory alloys depends strongly on the relationship between operating temperature and transformation temperatures, as described by the Clausius–Clapeyron relationship [42,43], the suitability of a given composition may vary under different service conditions. Therefore, alloy selection should consider both mechanical properties and the expected operational temperature range. Overall, the results demonstrate that relatively small compositional adjustments in CAB alloys can produce substantial changes in their mechanical and functional behavior.

### 3.2. ASTM F2516 Test Results

**Figure 10:**
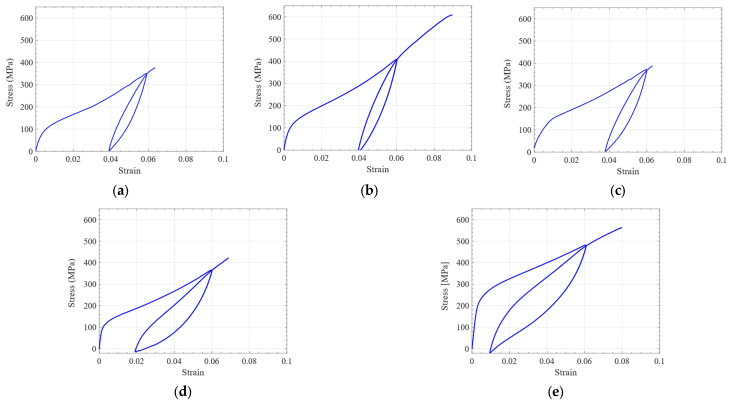
ASTM F2516 stress–strain curves for: (**a**) CAB4.4-AM, (**b**) CAB4.65-AM (**c**) CAB4.7-AM, (**d**) CAB4.45-A, (**e**) CAB4.76-A.

The ASTM F2516 [40] protocol, which is used for measuring the tensile properties of superelastic nickel-titanium (Nitinol) alloys, was applied to all alloys to evaluate superelastic performance. In this context, initial martensitic or mixed phase alloys showed a limited response under this protocol. In these cases, the resulting stress–strain curves did not show stable superelasticity and exhibited appreciable residual strains, characteristic of shape memory behavior, as shown in Figure 10a–c. The results for alloy CAB4.1-M (*M_s_* ≈ 70 °C) are not presented due data acquisition issues during testing.

In contrast, the austenitic phase alloys were expected to develop characteristic superelastic stress–strain. Thus, the CAB4.45-A alloy (Figure 10d) displayed a more defined transformation plateau and improved recovery, although moderate residual strain remained, while the CAB4.76-A alloy (Figure 10e) reached stresses exceeding 500 MPa and exhibited the most stable superelastic response, with minimal residual strain ($\varepsilon_{res}$ ≈ 0.0095).

Based on these results, CAB4.76-A was selected for the increasing-amplitude cyclic tests and subsequent numerical implementation in a UFP device. The selection was driven by its superior strength, transformation stability, and strain recovery capacity within the superelastic regime, rendering it an appropriate candidate for the development of resilient seismic devices. In addition to its mechanical performance, the selection of CAB4.76-A was guided by the expected operational temperature range of seismic devices in Chile (−10 °C to 45 °C). With *A_f_* ≈ −15 °C, this alloy ensures a fully austenitic phase throughout the service range, providing stable superelastic behavior. Although other investigate compositions may also exhibit superelasticity at temperatures sufficiently above their *A_f_;* only CAB4.76-A meets this criterion across the entire expected operational temperature range.

### 3.3. Increasing-Amplitude Half-Cycle Test Results

To characterize the mechanical behavior of CAB4.76-A under cyclic loading, the stress–strain response associated with each applied displacement level was evaluated. This analysis allows quantifying the material’s ability to withstand significant deformations without failure and estimating its potential for energy dissipation in structural applications.

**Figure 11:**
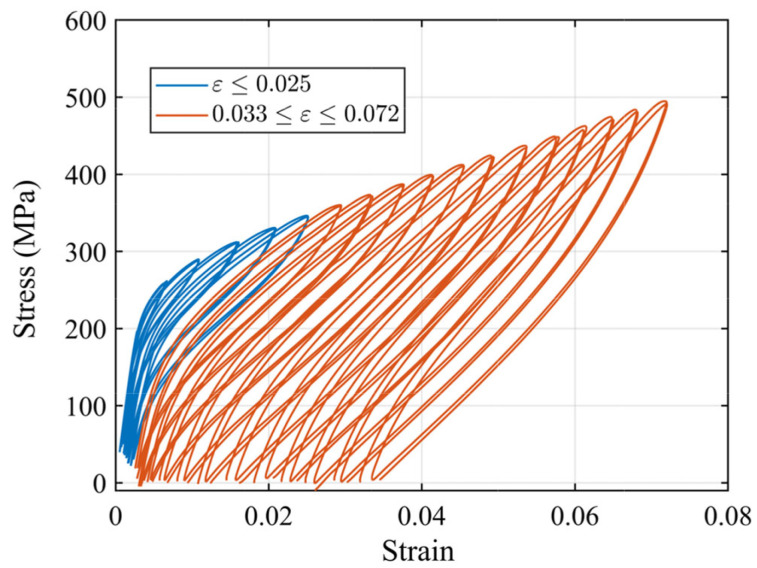
Stress–strain curve obtained in the cyclic test for specimen CAB4.76-A.

Figure 11 shows the evolution of the stress–strain behavior of the CAB4.76-A specimen under increasing cyclic loads. In the initial half-cycles, corresponding to strains up to approximately 0.025, the alloy exhibited a clearly superelastic response with nearly complete strain recovery upon unloading, consistent with the strain limits reported by [32]. Beyond this threshold, progressive accumulation of residual strain became evident, indicating gradual degradation of superelastic performance.

To further quantify cyclic behavior, the analysis incorporated complementary parameters. These include: (i) the energy dissipated per cycle, calculated as the area enclosed by the force–displacement hysteresis loops, which reflects the material’s ability to dissipate mechanical energy, and (ii) the strain recovery index (SRI), defined as the proportion of recovery relative to the maximum applied displacement, which is key for assessing superelastic behavior.

The strain recovery index (SRI) is used to quantify the elastic recovery capacity after each loading cycle and is defined in Equation (2).

(2) $$SRI=\frac{d_{max}-d_{i}}{d_{max}}$$

where, *d*_max_ corresponds to the maximum strain reached during the cycle, and *d*_*i*_ is the initial strain. Values of SRI close to 1 indicate high elastic recovery, whereas lower values reflect greater residual deformation.

**Figure 12:**
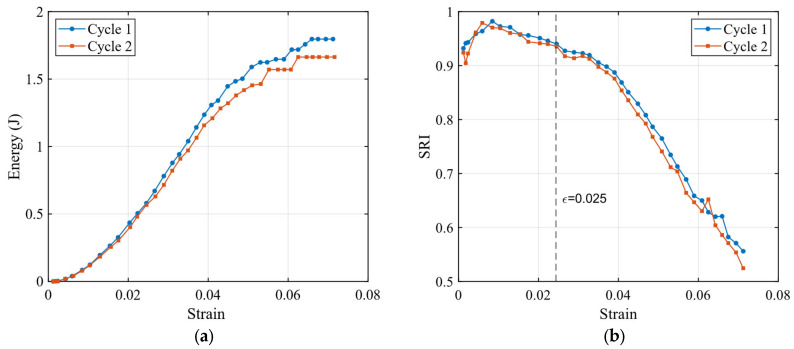
Complementary parameters evaluated for the CAB4.76-A alloy. (**a**) Energy dissipation, (**b**) strain recovery index (SRI).

Figure 12a shows that the dissipated energy increases continuously with strain, exhibiting an almost linear trend in the early stages and stabilizing at higher levels. Both half-cycles present a very similar response, indicating stable material behavior. The absence of significant discontinuities confirms that the system remains stable and that energy dissipation occurs primarily through reversible martensite–austenite phase transformations.

Figure 12b presents the strain recovery index (SRI), which remains high (SRI ≥ 0.95) up to a strain of approximately 0.025, indicating recovery above 95% within this range. For larger strains, SRI gradually decreases, marking the onset of functional degradation caused by residual strain accumulation, in agreement with experimental findings reported for Cu–Al–Be alloys in the literature [29,32]. These results support limiting subsequent numerical modeling to strains within the validated superelastic range (ε ≤ 0.025).

## 4. Numerical Finite Element Model (FEM)

### 4.1. Superelastic Model

A three-dimensional finite element model was implemented to simulate the superelastic behavior of the CAB4.76-A alloy, previously characterized experimentally. The analysis was carried out using the constitutive model for superelastic shape memory materials proposed by Auricchio et al. [44], available in the Static Structural module of ANSYS Workbench 2025R1 [45]. The model definition requires the specification of the elastic properties of austenite and martensite, including the elastic modulus (*E*) and Poisson’s ratio (ν), as well as the transformation strain associated with the martensitic transformation. In addition, the model requires the definition of the critical transformation stresses for the start (*σ*_S_^AS^) and finish (*σ*_F_^AS^) of the forward transformation (austenite to martensite) and start (*σ*_S_^SA^) and finish (*σ*_F_^SA^) of the reverse transformation (martensite to austenite), together with the maximum transformation strain ($\varepsilon_{L}$).

This phenomenological model allows reproducing the characteristic hysteretic curves of SMA materials in the quasi-static regime, without the need to explicitly model the microstructure. Its use has been widely validated in structural applications, making it an appropriate tool to capture the superelastic response of the material within the strain levels considered. It should be noted that the adopted constitutive model does not account for cyclic degradation or damage accumulation, which may influence long-term performance under repeated seismic loading.

### 4.2. Estimation of Model Parameters

Model parameters were calibrated using experimental monotonic and cyclic data for CAB4.76-A within the validated strain range. The analysis focuses on strains levels ε ≤ 0.025, corresponding to the superelastic regime with no significant residual strain as observed in the experimental results.

The specimen geometry was replicated in the numerical model using 10-node SOLID187 elements with a mesh size of 2 mm. Mesh refinement studies confirmed that further mesh reduction did not significantly affect global response, while maintaining reasonable computational efficiency.

One end of the specimen was fully restrained, and displacement control was applied at the opposite end. Nonlinear analysis was performed using incremental load steps with an initial time step of 0.015 s (minimum 0.001 s, maximum 0.04 s) to ensure convergence.

Calibration quality was assessed using the Root Mean Square Error (RMSE), defined in Equation (3).

(3) $$RMSE=\sqrt{\frac{1}{n}\sum_{i=1}^{n}{\left(\sigma_{i}^{exp}-\sigma_{i}^{num}\right)}^{2}},$$

where *σ*_exp_ and *σ*_num_ represent the experimental and numerical stress values, respectively, and n is the total number of data points considered in the comparison. This indicator quantifies the average deviation between both curves, with lower values indicating a better fit.

An RMSE of 7.79 MPa was obtained for the monotonic test and 51.77 MPa for the cyclic test. The low error in the monotonic case reflects excellent agreement between experimental and numerical results, whereas the higher value in the cyclic test is associated with the difficulty of accurately reproducing the accumulation of residual strains and the progressive degradation of superelasticity under repeated loading. These results indicate that the calibration performed is adequate within the superelastic range of interest. In particular, the low RMSE obtained for the monotonic case validates the ability of the model to reproduce the initial response of the material, while the overall behavior captured in the cyclic test confirms that the adopted parameters allow for a consistent representation of the hysteretic evolution without residual deformations. Consequently, calibration can be regarded as representative and provides a reasonable approximation of the material response within the calibrated strain range. However, the UFP simulations later presented in Section 5 should be interpreted within the scope of the calibrated strain range (ε ≤ 0.025) and are primarily intended to evaluate qualitative response trends, since cyclic degradation and fatigue mechanisms are not represented in the constitutive formulation.

The parameters used in the numerical simulations are presented in Table 3. For consistency with the experimental notation adopted in Table 2, the forward transformation stresses of the Auricchio model are related as σ_S_^AS^ ≡ σ_MS_ and σ_F_^AS^ ≡ σ_Mf_. The reverse transformation stresses (σ_S_^SA^ and σ_F_^SA^) correspond to the start and finish of the martensite-to-austenite transformation during unloading and are not reported in Table 2 because they were not extracted from the monotonic tests.

**Figure 13:**
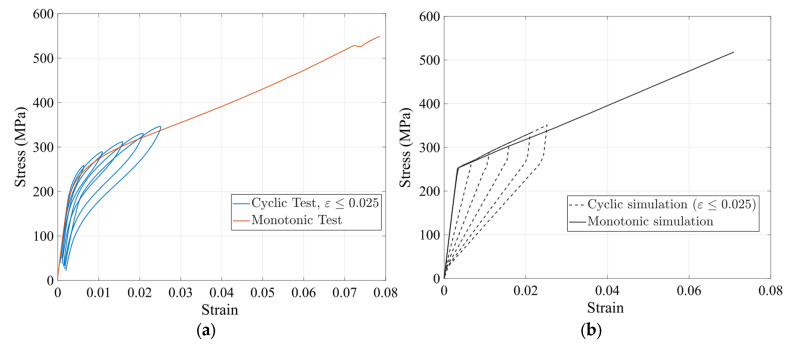
Comparison between monotonic and cyclic stress–strain curves for the CAB4.76-A alloy. (**a**) Experimental, (**b**) Numerical.

A variation is observed in the final stress of the forward transformation (*σ*_*F*_^*AS*^), whose value was 540 MPa for the monotonic test and 590 MPa for the cyclic test. This difference is attributed to the type of loading: while in the monotonic test the material is loaded only once up to fracture, in the cyclic case the repetition of half-cycles induces partial accumulation of martensite, generating a progressive hardening effect. As a result, higher stress is required to complete the phase transformation during subsequent cycles. This behavior can be observed in Figure 13, where (a) compares the experimental curves for both tests and shows that, for the same strain level, the stress in the cyclic test is higher. Figure 13b presents the numerical finite element simulation, which adequately reproduces this difference in behavior using the constitutive model implemented in ANSYS.

It is important to note that the constitutive model parameters were calibrated using experimental data obtained at T ≈ 10 °C. Since no thermomechanical coupling is incorporated in the present formulation, the transformation stresses and the resulting numerical predictions are valid only for the calibration temperature. Application of the model to other service temperatures would require recalibration of the transformation stress parameters using test data obtained at the corresponding temperature.

### 4.3. Model Validation

**Figure 14:**
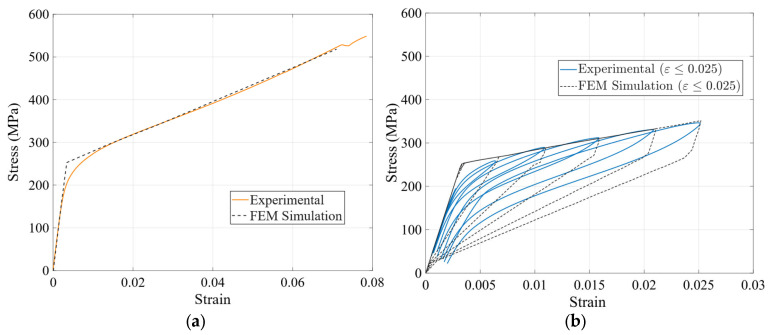
Experimental and numerical stress–strain curves for the CAB4.76-A alloy. (**a**) Monotonic test, (**b**) Cyclic test.

Numerical simulations were compared with experimental results for both monotonic and cyclic loading, as shown in Figure 14. A good agreement is observed in initial stiffness, transformation stress and overall response for both tests.

Table 4 summarizes the quantitative comparison between experimental and numerical results for different strain levels. The dissipated energy per cycle, the maximum force ($F_{max}$) and the secant modulus of elasticity ($E_{sec}$) are included as representative measures of cyclic behavior.

From these results, it is observed that the numerical model correctly reproduces the evolution of maximum force and secant modulus of elasticity with increasing strain, maintaining discrepancies below 5% for $F_{max}$ and on the order of 10–15% for $E_{sec}$. Thus, the model accurately reproduces the initial superelastic behavior but does not capture cumulative degradation effects arising from repeated cycling. This limitation stems from the constitutive formulation, which describes instantaneous superelastic response without incorporating internal damage variables or fatigue mechanisms. Accordingly, the model’s validity is limited to strains up to approximately 2.5%. Although larger strains, close to 7.7%, were experimentally achieved under monotonic loading, significant residual strain and recovery loss were observed beyond this threshold. Within the validated range, the model provides adequate predictive capability for stiffness and strength estimation. Nevertheless, its application to analyzes involving repeated cycling or fatigue would require more advanced constitutive models incorporating internal damage variables and cyclic degradation mechanisms.

## 5. Numerical Application to a U-Shape Flexural Plate (UFP)

A three-dimensional finite element model of a UFP manufactured from the selected CAB4.76-A alloy was developed to evaluate its structural response. The objective of this analysis is not to provide a validated design solution, but rather to explore the structural implications of using Cu–Al–Be SMAs in UFP configurations through numerical simulations based on experimentally calibrated material behavior.

The model was implemented in ANSYS using SOLID186 elements with a characteristic mesh size of 4 mm. The geometric properties of the UFP are as follows: total length of 100 mm, an effective length of 42 mm, a height of 60 mm, a diameter of 55 mm, a width of 60 mm, and a thickness of 5 mm.

**Figure 15:**
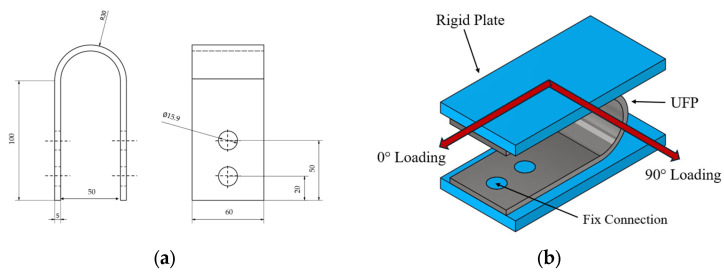
UFP device. (**a**) Geometry (units in mm), (**b**) Main loading directions.

Boundary conditions were applied through rigid plates at both ends of the device. The bottom plate was fully restrained, while the top plate was subjected to controlled displacement. The connection between the rigid plates and the UFP was modeled as bonded to represent a rigid mechanical joint. A frictionless contact condition was defined between the plate surfaces and the UFP. The aim of this simulation is to explore its behavior under two orthogonal directional loadings, since the curved arms of UFPs induce combined bending and torsion deformations. Figure 15 illustrates the UFP geometry and the two critical loading directions: 0° and 90°.

### 5.1. Applied Loading Protocol and Model Validity

**Figure 16:**
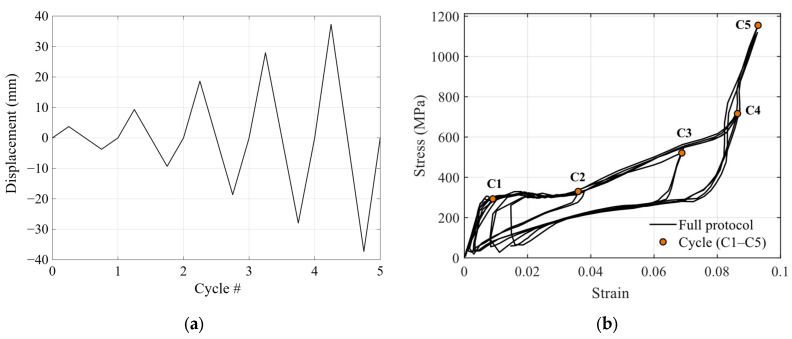
(**a**) Displacement history. (**b**) Stress–strain curve for the UFP subjected to 90° loading.

To determine the operational superelastic range of the UFP, a cyclic loading protocol with increasing displacement amplitudes was applied (Figure 16a), reaching a maximum displacement of ±37.3 mm.

Figure 16b presents the corresponding stress–strain response for the 90° loading direction, which was identified as the most critical orientation. Five characteristic cycle points (C1–C5) are highlighted, and their associated displacement, strain, and stress values are summarized in Table 5.

The results indicate that the superelastic model begins to exceed its validity range from the second cycle (C2) in the 90° loading direction, corresponding to a displacement amplitude of ±9.33 mm. This direction was identified as the most critical due to the shorter effective length of the device in that orientation (60 mm), which concentrates most of the curvature and produces localized increases in strain in the curved region of the UFP. At point C2, the material maintains strains close to 2.5%, corresponding to the experimentally validated superelastic range in which the material exhibited high strain recovery (SRI ≥ 0.95). At point C3, corresponding to a displacement amplitude of ±18.25 mm, local strains reached approximately 7%, approaching the maximum strain experimentally attained during monotonic and cyclic coupon tests without fracture. However, beyond this point, the numerical stress–strain curve (Figure 16b) shows pronounced stress increments which were not observed experimentally. This divergence reflects the limitations of the calibrated superelastic model outside the validated strain range. Consequently, a displacement amplitude of approximately ±9 mm is recommended as the practical upper limit for subsequent cyclic analyses. Within this range, the predicted response remains consistent with the experimentally validated behavior of the CAB4.76-A alloy, ensuring that the numerical simulations are confined to the calibrated superelastic regime and avoiding extrapolation into strain levels for which the constitutive model has not been validated.

### 5.2. Results and Discussion

**Figure 17:**
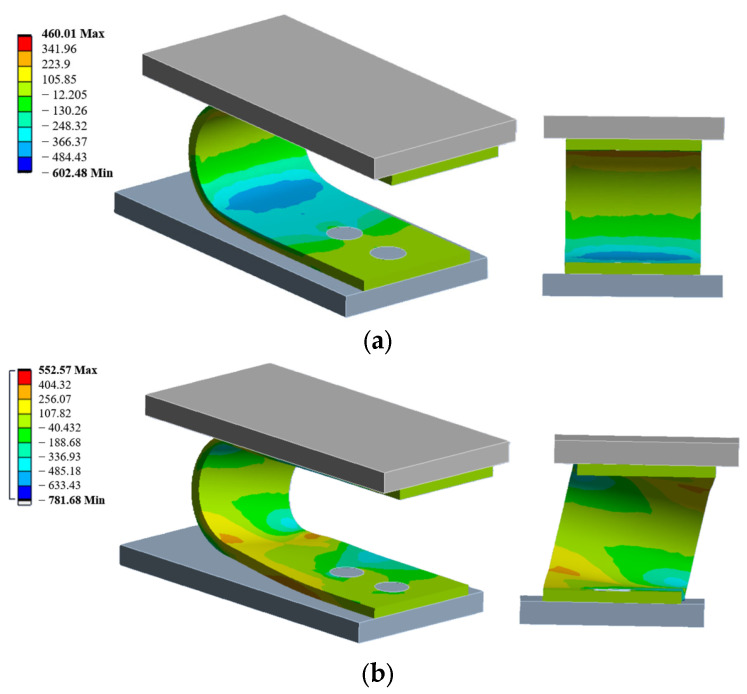
Maximum stresses in: (**a**) 0° loading; (**b**) 90° loading.

**Figure 18:**
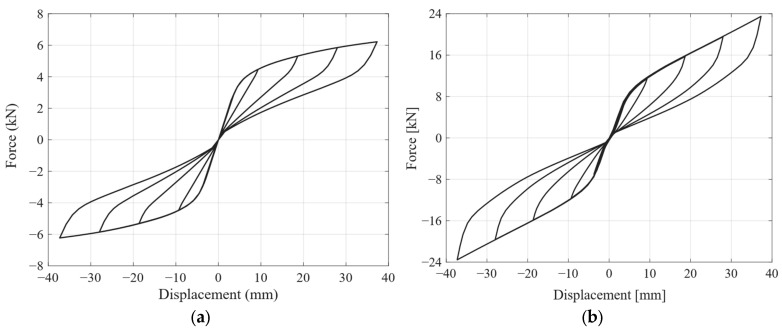
Force–displacement curves of a UFP loaded at (**a**) 0° and (**b**) 90°.

Figure 17 shows the overall deformation and stresses of the UFP when loaded in 0° and 90° directions, while Figure 18 presents the force–displacement curves obtained by applying the cyclic protocol with increasing amplitudes. In the 0° direction (Figure 18a), the last cycle reaches a maximum force of approximately 6.2 kN, whereas in the 90° direction (Figure 18b) forces up to 23.4 kN are recorded. This difference of 17.2 kN clearly reflects the significant impact of loading orientation on the structural response of the device.

The observed directional dependence is consistent with previous studies on steel UFPs, where geometric effects govern stiffness and strength [6]. However, in the case of SMA-based devices, this effect is coupled with the material’s superelastic response, which influences both energy dissipation and recentering capability. Compared to conventional steel dampers, the SMA-based UFP is expected to exhibit reduced residual deformation, although this comparison was not directly validated at the device level in the present study.

The observed behavior is primarily attributed to geometric effects. In the 90° direction, the curved arms experience combined bending and torsion, resulting in higher stiffness and force capacity. In the 0° direction, the deformation mechanism is more flexural-dominated, leading to lower force demand. These findings emphasize that UFP orientation must be explicitly considered in seismic design applications, particularly when multidirectional loading is expected.

**Figure 19:**
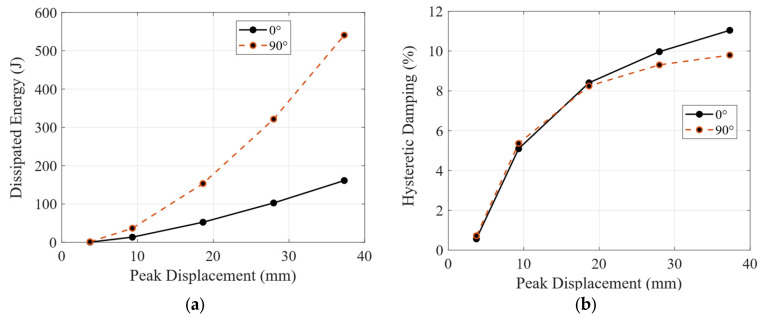
Energy Dissipation capacity. (**a**) Dissipated energy per cycle, (**b**) Hysteretic damping.

Due to the differences in maximum strength, the loading orientation also influences the total dissipated energy of the system. Figure 19 shows the evolution of the energy dissipated per cycle as a function of peak displacement. In the 0° direction, the dissipated energy reaches a maximum value of 161.2 J, whereas in the 90° direction this value increases to 540.7 J, which is more than three times higher.

The equivalent hysteretic damping ratio (*ξ*_hyst_) was also determined from the relationship between dissipated energy and elastic strain energy in each cycle, useful for earthquake engineering [2,6,46,47,48,49]. Despite the difference in total dissipated energy, the hysteretic damping of the UFP remains close to 10% in both directions. This indicates that, although absolute energy capacity differs, the relative damping efficiency of the device remains consistent. These results suggest that Cu–Al–Be UFP devices can achieve reasonable hysteretic damping while maintaining self-centering capability within the validated strain range. Accordingly, the numerical results should be interpreted as representative of the response within the validated strain range. Predictions associated with long-term cyclic degradation, fatigue life, and progressive loss of energy dissipation capacity remain outside the scope of the adopted constitutive model and require further investigation using advanced SMA formulations.

For seismic design purposes, UFP stiffness and yield force are critical parameters [2]. The initial stiffness in each loading direction was computed from the slope of the force–displacement curve in its initial linear segment. These numerical results were then compared to the equations proposed by González et al. [6]. It is important to note that these expressions were originally developed for steel UFPs with different geometries and their application to SMA UFPs may therefore be limited. The stiffness (K) in both directions was estimated using the following expressions:

(4) $$K^{\left(0{}^{\circ}\right)}=\frac{16EB}{27\pi }{\left(\frac{t}{D}\right)}^{3}$$

(5) $$K^{\left(90{}^{\circ}\right)}=C_{k}{\cdot K}^{\left(0{}^{\circ}\right)}$$

where E is the modulus of elasticity, that for the CAB4.76-A was estimated as 79 GPa for the numerical simulations. *B* is the width of the UFP equal to 60 mm, *t* is the thickness equal to 5 mm, and *D* is the diameter equal to 55 mm.

The yield force ($F_{y}$) in both directions was computed using the following expressions:

(6) $$F_{y}^{\left(0{}^{\circ}\right)}=f_{y}\left(\frac{Bt^{2}}{6R+t}\right)$$

(7) $$F_{y}^{\left(90{}^{\circ}\right)}=C_{F_{y}}\cdot F_{y}^{\left(0{}^{\circ}\right)}$$

where $f_{y}$ is the yield stress that in the case of SMA materials corresponds to the start stress of the superelastic transformation (*σ*_*Ms*_), which for the CAB4.76-A was estimated as 250 MPa, R is the radius of the UFP, which is equal to 27.5 mm, and $C_{k}$ and $C_{F_{y}}$ are correction factors that depend on the height (H) to thickness (t) ratio, as shown in Equation (8). In this case, the ratio (H/t) is equal to 12 and the parameters used in the equation are presented in Table 6.

(8) $$C_{k}\;\;or\;\;C_{F_{y}}=a_{0}+a_{1}\frac{H}{t}+a_{2}{\left(\frac{H}{t}\right)}^{2}$$

Table 7 summarizes the results obtained from the comparison between the expressions proposed by González et al. [6] and those obtained from the numerical simulation. For the initial stiffness, the relative error in the 0° loading direction is approximately 18%, whereas in the 90° direction the error decreases to about 4%. The improved agreement in the 90° direction suggests that, at least at a global level, the coupled bending–torsion response that governs the structural behavior of the UFP under this loading orientation is captured. Regarding the yield force, larger discrepancies are observed, with relative errors of 36% and 30% in the 0° and 90° directions, respectively. These differences indicate that the formulations, although useful for preliminary estimations, do not fully reproduce the response of UFP devices manufactured from superelastic CAB alloys.

Consequently, the observed discrepancies cannot be attributed solely to geometric effects or uncertainty in the correction factors, but also to the different deformation mechanisms underlying steel and SMA-based devices. The present results suggest that the existing relationships may provide reasonable preliminary estimates of stiffness and strength for CAB UFPs; however, they should not be regarded as fully predictive design equations. While refined correction factors could potentially improve agreement for the specific geometry and alloy investigated in this study, a more rigorous formulation explicitly accounting for the thermo-mechanical behavior of shape memory alloys would likely be required for generalized application. Therefore, existing expressions are used primarily as a benchmark for comparison rather than as validated design equations. Dedicated experimental testing and broader parametric studies involving different geometries, alloy compositions, temperature characterization and loading conditions are required to establish reliable formulations for SMA-based UFP devices.

## 6. Conclusions

This study presented an experimental characterization and numerical assessment of CAB shape memory alloys for potential application in UFP seismic dampers. Based on the results presented in the paper, the following conclusions can be drawn:

The experimental characterization demonstrated that the mechanical and functional response of Cu–Al–Be SMAs is highly sensitive to chemical composition, particularly to small variations in Be content, which produced significant shifts in transformation temperatures and directly affected the superelastic behavior of the alloys.

Among the six evaluated alloys, CAB4.76-A exhibited the best overall performance for structural applications, achieving the highest elastic modulus (69.7 GPa), transformation stress (239 MPa), and ultimate stress (525 MPa), together with stable pseudoelastic behavior and residual strains below 1% under the ASTM F2516 cyclic loading.

Increasing-amplitude cyclic tests demonstrated that CAB4.76-A maintained stable superelastic behavior within strains up to approximately ε ≤ 0.025, with a strain recovery index (SRI) greater than 0.95, corresponding to recovery levels above 95%. Beyond this limit, progressive residual strain accumulation and degradation of superelasticity were observed. Owing to its excellent recovery capacity within the validated strain range, high transformation stresses, and an austenite finish temperature (*A_f_* ≈ −15 °C) well below the anticipated service temperature range (−10 °C to 45 °C), CAB4.76-A was identified as the most suitable alloy among those investigated for seismic applications requiring both high load-bearing capacity and self-centering capability.

The phenomenological finite element model based on the Auricchio constitutive formulation successfully reproduced the experimental response of the alloy within the validated superelastic range, with good agreement in stiffness, transformation stress, and hysteretic response.

The numerical assessment of the UFP revealed a strong dependence on loading orientation. The 90° direction reached maximum forces of 23.4 kN and dissipated energies of 540.7 J, whereas the 0° direction reached only 6.2 kN and 161.2 J, respectively, demonstrating the significant influence of coupled bending–torsion effects on the structural response. Despite these differences, the equivalent hysteretic damping ratio remained close to 10% in both cases, demonstrating stable damping efficiency.

Comparison with analytical formulations originally developed for steel UFPs revealed that, although these expressions can provide reasonable preliminary estimates, they do not fully capture the response of SMA-based devices due to the fundamentally different thermomechanical response and energy dissipation mechanisms associated with stress-induced phase transformations. The observed discrepancies highlight the need for analytical models specifically formulated for superelastic materials and validated through experimental testing of full-scale devices.

Overall, the results confirm that Cu–Al–Be SMAs, particularly CAB4.76-A, are promising low-cost alternatives for self-centering seismic energy dissipation devices due to their high recoverable strain, stable hysteretic response, and energy dissipation capacity in the reference temperature used in this study. Nevertheless, further experimental validation at the device scale and constitutive models incorporating cyclic degradation and fatigue effects are still required.

## Figures and Tables

**Table 1:** Chemical composition and transformation temperatures of the samples.

Sample	Cu [wt%]	Al [wt%]	Be [wt%]	*M_s_* [°C]	*M_f_* [°C]	*A_s_* [°C]	*A_f_* [°C]	Phase at T = 10 °C
CAB4.10-M	87.89	11.70	0.41	70.14	40.61	91.15	144.01	Martensite
CAB4.40-AM	87.86	11.70	0.44	25.69	5.11	30.22	68.87	Martensite + Austenite
CAB4.65-AM	87.545	11.99	0.465	21.33	7.24	20.23	29.99	Martensite + Austenite
CAB4.70-AM	88.94	10.59	0.47	6.94	−15.21	3.46	23.3	Martensite + Austenite
CAB4.45-A	87.605	11.95	0.445	6.62	−11.19	2.37	16.81	Austenite
CAB4.76-A	87.534	11.99	0.476	−23.48	−40.88	−29.91	−15.4	Austenite

**Table 2:** Experimental mechanical properties for the alloys.

Sample	Al [wt%]	Be [wt%]	*E_m_* (MPa)	*E_A_* (MPa)	*E_y_* (MPa)	$\sigma_{s}$($\sigma_{Ms}$) (MPa)	$\sigma_{f}$($\sigma_{Mf}$ ) (MPa)	$\varepsilon_{s}$($\varepsilon_{Ms}$)	$\varepsilon_{f}$($\varepsilon_{Mf})$
CAB4.10-M	11.70	0.41	39,452	-	2159	71	278	0.0026	0.0718
CAB4.40-AM	11.70	0.44	27,923	-	2280	85	385	0.0040	0.0943
CAB4.65-AM	11.99	0.465	30,543	-	5268	98	459	0.0047	0.0663
CAB4.70-AM	10.59	0.47	16,894	-	2947	71	349	0.0039	0.0873
CAB4.45-A	11.95	0.445	-	64,652	3928	(139)	(385)	(0.0054)	(0.0600)
CAB4.76-A	11.99	0.476	-	69,667	3612	(239)	(525)	(0.0060)	(0.0724)

**Table 3:** Parameters used for the superelastic constitutive model for the CAB4.76-A alloy in the numerical simulations. In this case σ_S_^AS^ ≡ σ_MS_ and σ_F_^AS^ ≡ σ_Mf_ for comparison with Table 2.

Parameter	Symbol	Monotonic	Cyclic
Elastic modulus (austenitic phase)	*E*	79,000 MPa	79,000 MPa
Poisson’s ratio	*ν*	0.30	0.30
Start stress of forward transformation	*σ*_S_^AS^	250 MPa	250 MPa
Finish stress of forward transformation	*σ*_F_^AS^	540 MPa	590 MPa
Start stress of reverse transformation	*σ*_S_^SA^	270 MPa	270 MPa
Finish stress of reverse transformation	*σ*_F_^SA^	20 MPa	20 MPa
Maximum pseudoelastic strain	*ε* *L*	0.077	0.077
Smooth transition parameter	*α*	0.1	0.1

**Table 4:** Comparison between experimental and numerical results for the CAB4.76-A alloy.

Strain ***ε***	Dissipated Energy (J)		$F_{max}$		$E_{Sec}$(MPa)	
	Exp.	Num.	Exp.	Num.	Exp.	Num.
0.0024	0.00299	0.00025	4.86	4.11	86,936	77,444
0.0065	0.03910	0.44978	6.46	6.71	45,167	62,713
0.0100	0.12347	0.91325	7.24	7.22	28,565	58,152
0.0155	0.26412	1.3826	7.80	7.76	21,140	54,806
0.0200	0.43508	1.7780	8.26	8.32	17,091	50,984
0.0250	0.39889	1.9714	8.66	8.78	12,688	43,683

**Table 5:** Summary of the characteristic points of the applied loading protocol.

Point	Displacement [mm]	Strain (%)	Stress [MPa]
C1	3.73	0.9	293.3
C2	9.33	3.6	330.0
C3	18.65	6.9	521.3
C4	27.98	8.6	716.5
C5	37.30	9.3	1155.0

**Table 6:** Coefficient values for the equations describing $C_{k}$ and $C_{F_{y}}$ proposed by [6].

	C_K_			C_Fy_		
B/t	$a_{0}$	$a_{1}$	$a_{2}$	$a_{0}$	$a_{1}$	$a_{2}$
12.5	−1.31	0.53	−0.018	−1.01	0.56	−0.023

**Table 7:** Comparison between initial stiffness and yield force obtained theoretically and from numerical simulation.

Direction	Stiffness [kN/mm]			Yield Force [kN]		
	Theoretical	Simulation	Relative Error [%]	Theoretical	Simulation	Relative Error [%]
0°	0.67	0.82	18	2.21	1.63	36
90°	1.65	1.58	4	5.29	4.08	30

## Data Availability

The original contributions presented in this study are included in the article. Further inquiries can be directed to the corresponding author.
